# Supplementary material for: The Relationship between Social Environmental Factors and Motor Performance in 3- to 12-Year-Old Typically Developing Children: A Systematic Review
Source: Int J Environ Res Public Health. 2021 Jul 14;18(14):7516. doi: 10.3390/ijerph18147516 (PMC8306533; doi:10.3390/ijerph18147516)
Supplement: Supplementary file 1 [file ijerph-18-07516-s001.zip › ijerph-1279031-supplementary.pdf]

Supplementary Materials

Table S1. Electronic databases and search strings according to the rules of each database.

Database	Search string
MEDLINE	("Child"[Mesh] OR child*[tiab] OR youth[tiab] OR teen*[tiab] OR school*[tiab] OR kids[tiab] OR preschool*[tiab]) AND ("Social Environment"[Mesh] OR social correlate*[tiab] OR social determinant*[tiab] OR social support*[tiab] OR social relat*[tiab] OR "Family"[Mesh] OR famil*[tiab] OR parent*[tiab] OR grandparent*[tiab] OR grandmother*[tiab] OR grandfather*[tiab] OR mother*[tiab] OR father*[tiab] OR maternal*[tiab] OR paternal*[tiab] OR parent-child*[tiab] OR mother-child*[tiab] OR father-child*[tiab] OR sibling*[tiab] OR brother*[tiab] OR sister*[tiab] OR birth order*[tiab] OR single-parent*[tiab] OR two-parent*[tiab] OR stepfamil*[tiab] OR stepfather*[tiab] OR stepmother*[tiab] OR childrearing [tiab] OR child-rearing [tiab] OR "Friends"[Mesh] OR friend*[tiab] OR playmate*[tiab] OR peer*[tiab] OR "School Teachers"[Mesh] OR teacher*[tiab] OR teacher-child*[tiab] OR coach*[tiab] OR trainer*[tiab]) AND ("Motor Skills"[Mesh] OR motor*[tiab]) NOT ("animals"[MeSH] NOT "humans"[MeSH]) NOT ("Review" [Publication Type]) NOT ("Cerebral Palsy"[Mesh] OR Cerebral palsy [tiab] OR "Autism Spectrum Disorder"[Mesh] OR autism [tiab] OR developmental coordination disorder* [tiab])
EMBASE	(('child'/exp OR ‘child*’:ab,ti OR ‘youth’:ab,ti OR ‘teen*’:ab,ti OR ‘school*’:ab,ti OR ‘kids’:ab,ti OR ‘preschool*’:ab,ti) AND ('social environment'/exp OR ‘social correlate*’:ab,ti OR ‘social determinant*’:ab,ti OR ‘social support*’:ab,ti OR ‘social relat*’:ab,ti OR 'family'/exp OR ‘famil*’:ab,ti OR ‘parent*’:ab,ti OR ‘grandparent*’:ab,ti OR ‘grandmother*’:ab,ti OR ‘grandfather*’:ab,ti OR ‘mother*’:ab,ti OR ‘father*’:ab,ti OR ‘maternal*’:ab,ti OR ‘paternal*’:ab,ti OR ‘parent-child*’:ab,ti OR ‘mother-child*’:ab,ti OR ‘father-child*’:ab,ti OR ‘sibling*’:ab,ti OR ‘brother*’:ab,ti OR ‘sister*’:ab,ti OR ‘birth order*’:ab,ti OR ‘single-parent*’:ab,ti OR ‘two-parent*’:ab,ti OR ‘stepfamil*’:ab,ti OR ‘stepfather*’:ab,ti OR ‘stepmother*’:ab,ti OR ‘childrearing’:ab,ti OR ‘child-rearing’:ab,ti OR 'friend'/exp OR ‘friend*’:ab,ti OR ‘playmate*’:ab,ti OR ‘peer*’:ab,ti OR 'teacher'/exp OR ‘teacher*’:ab,ti OR ‘teacher-child*’:ab,ti OR ‘coach*’:ab,ti OR ‘trainer*’:ab,ti) AND ('motor performance'/exp OR ‘motor*’:ab,ti) NOT ('animal'/exp NOT 'human'/exp) NOT ('review'/exp) NOT ('cerebral palsy'/exp OR 'autism'/exp OR 'developmental coordination disorder'/exp OR 'disabled person'/exp)) AND [english]/lim AND [2000-2020]/py

Database	Search string
PsycINFO	TI (child* OR youth OR teen* OR school* OR kids OR preschool*) OR AB (child* OR youth OR teen* OR school* OR kids OR preschool*)<br>AND DE ("Home Environment" OR "Classroom Environment" OR "School Environment" OR "Family Members" OR "Family Relations" OR "Family Structure" OR "Stepfamily" OR "Peers" OR "Peer Relations" OR "Teachers") OR TI (social correlate* OR social determinant* OR social support* OR social relat* OR famil* OR parent* OR grandparent* OR grandmother* OR grandfather* OR mother* OR father* OR maternal* OR paternal* OR parent-child* OR mother-child* OR father-child* OR parental* OR sibling* OR brother* OR sister* OR birth order* OR single-parent* OR two-parent* OR stepfamil* OR stepfather* OR stepmother* OR childrearing OR child-rearing OR friend* OR playmate* OR peer* OR teach* OR teacher-child* OR coach* OR trainer*) OR AB (environment* OR social correlate* OR social determinant* OR social support* OR social relat* OR famil* OR parent* OR grandparent* OR grandmother* OR grandfather* OR mother* OR father* OR maternal* OR paternal* OR parent-child* OR mother-child* OR father-child* OR sibling* OR brother* OR sister* OR birth order* OR single-parent* OR two-parent* OR stepfamil* OR stepfather* OR stepmother* OR childrearing OR child-rearing OR friend* OR playmate* OR peer* OR teacher* OR teacher-child* OR coach* OR trainer*)<br>AND DE ("Motor Skills") OR TI (motor*) OR AB (motor*)<br>NOT DE ("Literature Review")<br>NOT DE ("Cerebral Palsy" OR "Autism Spectrum Disorders" OR "Dyspraxia")
ERIC	TI (child* OR youth OR teen* OR school* OR kids OR preschool*) OR AB (child* OR youth OR teen* OR school* OR kids OR preschool*)<br>AND DE ("Family Environment" OR "Classroom Environment" OR "Family Structure" OR "Family Characteristics" OR "Family Relationship" OR "Peer Relationship" OR "Friendship" OR "Teacher Characteristics") OR TI (environment* OR social correlate* OR social determinant* OR social support* OR social relat* OR famil* OR parent* OR grandparent* OR grandmother* OR grandfather* OR mother* OR father* OR maternal* OR paternal* OR parent-child* OR mother-child* OR father-child* OR parental* OR sibling* OR brother* OR sister* OR birth order* OR single-parent* OR two-parent* OR stepfamil* OR stepfather* OR stepmother* OR childrearing OR child-rearing OR friend* OR playmate* OR peer* OR teach* OR teacher-child* OR coach* OR trainer*) OR AB (environment* OR social correlate* OR social determinant* OR social support* OR social relat* OR famil* OR parent* OR grandparent* OR grandmother* OR grandfather* OR mother* OR father* OR maternal* OR paternal* OR parent-child* OR mother-child* OR father-child* OR parental* OR sibling* OR brother* OR sister* OR birth order* OR single-parent* OR two-parent* OR stepfamil* OR stepfather* OR stepmother* OR childrearing OR child-rearing OR friend* OR playmate* OR peer* OR teach* OR teacher-child* OR coach* OR trainer*)<br>AND DE (Psychomotor Skills") OR TI (motor*) OR AB (motor*)<br>NOT DE ("Literature Reviews")<br>NOT DE ("Cerebral Palsy" OR "Autism")

Table S2. Quality assessment of the included studies.

Study	Study Participation	Study Attrition	Prognostic Factor Measurement	Outcome Measurement	Study Confounding	Statistical Analysis and Reporting
Barnett et al., 2019	High	High	Low	Low	Low	Low
Jensen et al., 2019	High	N.A.	Low	Low	Moderate	Low
Zeng et al., 2019	Moderate	N.A.	Low	Low	High	Low
Simcock et al., 2018	Low	N.A.	Low	Low	Low	Low
Luz et al., 2018	Low	N.A.	Low	Low	Low	Low
Wolf & McCoy, 2019	Low	N.A.	Low	Low	Low	Low
Sartori et al., 2017	Moderate	N.A.	Moderate	Low	Low	Low
True et al., 2017	Low	N.A.	Low	Low	Low	Low
Comuk-Balci et al., 2016	High	N.A.	Low	Low	High	Moderate
Hua et al., 2016	Moderate	N.A.	Low	Low	Low	Low
Bindman et al., 2014	Low	N.A.	Low	Low	Low	Low
Cao et al., 2014	Moderate	High	Low	Low	Low	Low
Barnett et al., 2013	Low	N.A.	Low	Low	Low	Low
Wu et al., 2012	Moderate	N.A.	Low	Low	Low	Low
Taverna et al., 2011	Low	N.A.	Low	Low	Low	Low
Cools et al., 2011	Low	N.A.	Low	Low	Low	Low
Livesey et al., 2011	Low	N.A.	Low	Low	Low	Low
Lung et al., 2011	High	N.A.	Low	Low	Moderate	Low
Krombholz, 2006	Moderate	N.A.	High	Low	High	High
Fabes et al., 2003	Low	N.A.	Low	Low	Low	Low
Lejarraga et al., 2002	High	N.A.	Low	Low	Moderate	Low
Peyre et al., 2019	Low	Moderate	Low	Low	Low	Low
Kumar et al., 2016	High	N.A.	Moderate	Low	Low	High
Giagazoglou et al., 2011	Low	N.A.	High	Low	Low	Low
Lin et al., 2020	Low	N.A.	Low	Low	Low	Low
de Oliveira & Jackson, 2017	Low	N.A.	Low	Low	Moderate	Low
Herry et al., 2007	Low	N.A.	Low	Moderate	High	Moderate
Moller, Forbes-Jones, & Hightower, 2008	Low	Low	High	Low	Low	Low
Moller, Forbes-Jones, Hightower, et al., 2008	Low	Low	High	Low	Low	Low
Chaves et al., 2015	Low	N.A.	Low	Low	Low	Low
Lin & Li, 2019	Low	N.A.	Low	Low	Low	Low

Note. Quality assessment was performed with the Quality of Prognosis Studies in Systematic Reviews (QUIPS) tool (Hayden et al., 2006, 2013). Abbreviations: N.A. = not applicable.
